# Decreased long non-coding RNA MTM contributes to gastric cancer cell migration and invasion via modulating MT1F

**DOI:** 10.18632/oncotarget.22126

**Published:** 2017-10-26

**Authors:** Zhenghua Lin, Sanchuan Lai, Xingkang He, Wei Zhuo, Lan Wang, Jianmin Si, Shujie Chen

**Affiliations:** ^1^ Department of Gastroenterology, Sir Run Run Shaw Hospital, School of Medicine, Zhejiang University, Hangzhou 310020, Zhejiang Province, China; ^2^ Institute of Gastroenterology, Zhejiang University, Hangzhou 310016, Zhejiang Province, China; ^3^ Department of Cell Biology and Program in Molecular Cell Biology, Zhejiang University School of Medicine, Hangzhou 310058, Zhejiang Province, China

**Keywords:** long noncoding RNA, MTM, cell invasion, MT1F, gastric cancer

## Abstract

The role of long non-coding RNAs (lncRNA) on gastric cancer (GC) are an emerging field. Here, we focused on a cancer-related lncRNA MTM and tried to explore its correlation with the development of GC. The expression of MTM was detected by qRT-PCR in GC cell lines and tissues. The relationship between MTM level and clinicopathological factors was then analyzed. Cell biological assays with overexpression or co-transfection approaches were examined to probe the functional relevance of this lncRNA and its potential targets. The results showed that MTM expression was significantly lower in GC cell lines and tissues, and closely correlated with lymphatic metastasis, invasive depth, tumor staging and overall survival. Overexpression of MTM significantly inhibited GC cell migration and invasion, suppressed cell proliferation and induced cell apoptosis. In addition, we found a positive correlation between the expression level of MTM and MT1F both in cell and tissue samples. MT1F overexpression decreased GC cell migration and invasion, while knockdown of MT1F restored cell migration and invasion in MTM-overexpressing GC cells, suggesting MT1F as a key target of MTM. Conclusively, abnormal decreased expression of MTM was observed in human GC, which might contribute to gastric carcinogenesis by modulating MT1F expression.

## INTRODUCTION

Gastric cancer (GC) is one of the most common malignancies of the digestive system and ranks the third leading cause of cancer-related death worldwide, with more than 723,000 patients dying from GC annually [1]. Though early detection for GC can improve the 5-year survival rates to 96% [2], the majority of GC cases present at an advanced stage upon initial diagnosis, and the prognosis of these patients is still disappointing. Gastric carcinogenesis is a multistep process resulted from the accumulation of numerous genetic and epigenetic aberrations. The molecular mechanisms underlying the process remain elusive. It is important to elucidate these mechanisms and identify novel molecular targets for early diagnosis and developing effective therapies for GC.

It has now been widely accepted that the overwhelming majority of human genome is transcribed into long non-coding RNAs (lncRNAs), defined as a class of non-coding RNA more than 200bp in length [3, 4]. Over the last decade, progresses about cancer-related lncRNAs have been achieved. Some excellent examples include oncogenic property of HOTAIR in breast cancer [5], HULC in liver cancer [6], MALAT1 in lung cancer [7], and tumor suppressive function of lncRNA PTENP1 in prostate cancer [8]. Accumulating evidence suggests that dysregulation of some lncRNAs have clinical significances in GC [9-13]. By interaction with DNA, RNA and proteins, lncRNAs play versatile roles in gastric carcinogenesis [14]. Nonetheless, the functional involvement of lncRNAs in GC has not been extensively studied.

MTM, also known as metallothionein 1D Pseudogene (MT1DP), is located at chromosome 16q13 and a member of metallothionein (MT) family (MT1 to MT4) [15]. Recently, Yu et al [16] have found that MTM exerted a tumor suppressive role in liver cancer cells and was negatively regulated by YAP (Yes associated protein) and Runx2 (Runt related transcription factor 2). However, the understanding of MTM is rather limited and the correlation of MTM with GC and its underlying mechanisms have yet not been reported. In the current study, we assessed the expression level of MTM in human GC tissues and cell lines and further investigated the functional relevance of MTM in GC.

## RESULTS

### Decreased expression of MTM in human GC

**Figure 1:**
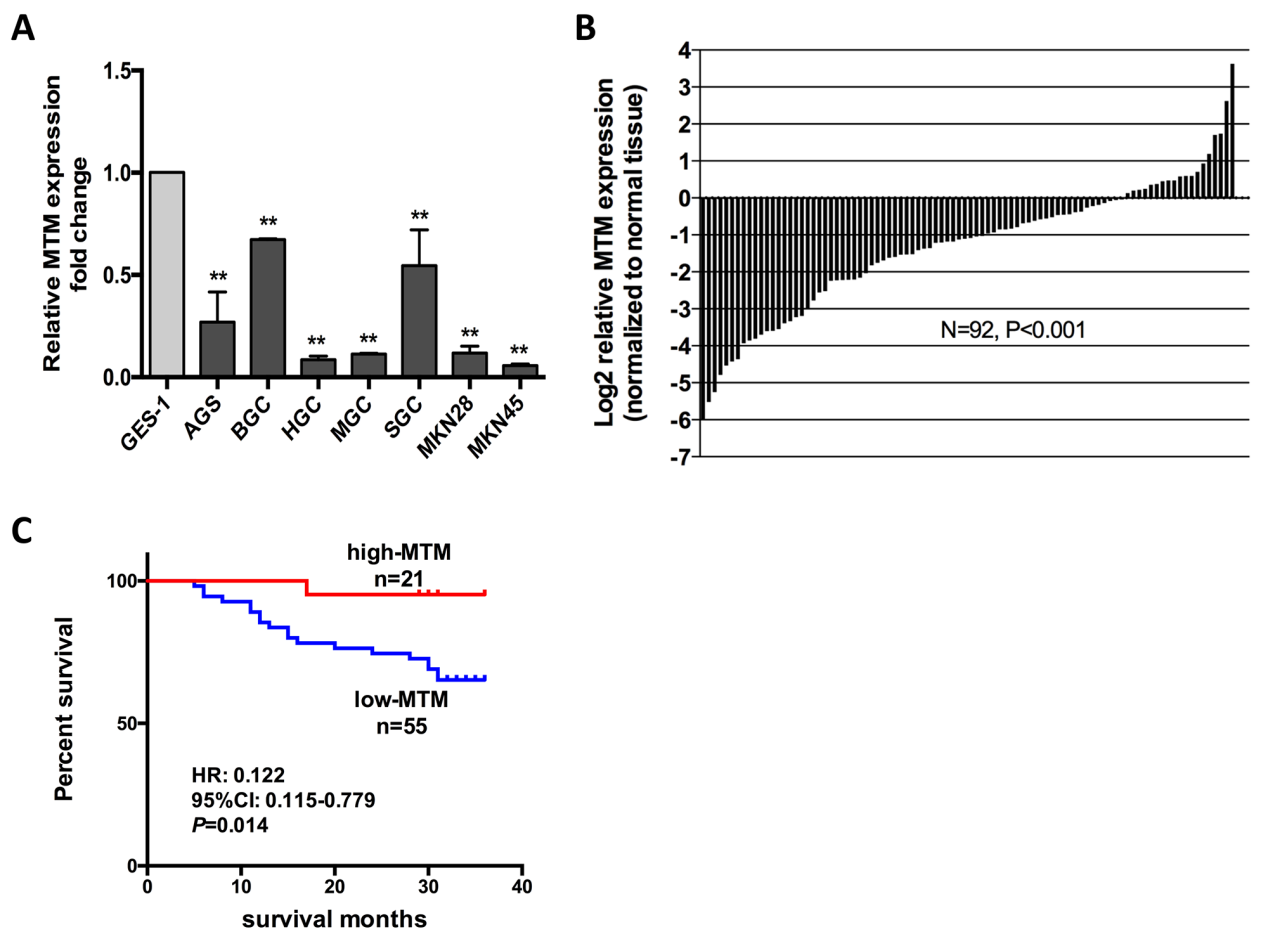
MTM expression is down regulated in human gastric cancer (GC) and related to patient survival **(A)** Expression of MTM in 7 GC cell lines and GES-1. Expression levels are normalized to GES-1. Data were presented as the mean ± SD (n=3, ^**^P<0.01). **(B)** MTM expression was significantly decreased in GC tissues relative to their corresponding normal gastric mucosa (NGM) tissues in 92 GC patients. Data are shown as log2-fold change to matching NGM tissues. **(C)** Patients with lower MTM expression showed decreased overall survival compared with patients with higher MTM expression.

We proceeded to validate the existence of MTM in GC cell lines by sequencing the RT-PCR products, and found that its sequence was consistent with that from the genome database (NR_027781.1; http://www.ncbi.nlm.nih.

gov/nuccore/239835749/). Then, we determined differential expression of MTM in GC cell lines and tissues. qRT-PCR revealed that MTM was downregulated in all 7 GC cell lines compared to GES-1 (Figure 1A). Subsequently, matched adjacent normal gastric mucosa (NGM) -GC tissue pairs from 92 GC patients were interrogated for MTM expression by qRT-PCR. MTM expression was significantly reduced relative to NGM in the majority of GC patients (73/92, average fold change 0.398, *P*<0.01, Figure 1B).

### Correlation between MTM expression and clinicopathological features in GC

Then, we examined whether MTM expression correlated with the clinicopathological features of GC patients. The expression level of MTM in tumor tissues from 92 GC patients was evaluated using qRT-PCR. Decreased MTM expression correlated with advanced tumor progression, which was characterized as increased lymphatic metastasis (*P*<0.01), enhanced invasive depth (*P*<0.05) and more advanced tumor staging (*P*<0.05). No correlation was found between MTM expression and other parameters such as age, gender, tumor size, differentiation or distant metastasis (Table 1).

**Table 1:** Correlation between MTM expression and clinicopathological features in gastric cancer

Clinicopathological features	No. of patients (N=92)	MTM expression evaluated by qRT-PCR	*P* value
**Age**			0.068
<60	37	0.0041	
≥60	55	0.0039	
**Gender**			0.487
Male	64	0.0038	
Female	28	0.0042	
**Tumor size**			0.113
<5cm	44	0.0049	
≥5cm	48	0.0031	
**Differentiation**			0.190
Poor	57	0.0031	
Moderate/High	35	0.0054	
**Distant metastasis**			0.313
M0	85	0.0042	
M1	7	0.0017	
**Lymphatic metastasis**			0.003^**^
No	36	0.0061	
Yes	56	0.0026	
**Invasive depth**			0.016^*^
T1	18	0.0061	
T2	12	0.0075	
T3	7	0.0015	
T4	55	0.0028	
**Stage (TNM)**			0.011^*^
I	24	0.0076	
II	15	0.0028	
III	46	0.0028	
IV	7	0.0017	

The results are expressed as the mean of 2^-ΔCT^. ^*^P<0.05; ^**^P<0.01.

We further investigated the relationship between MTM expression and prognosis of GC patients. Kaplan–Meier survival analysis showed that decreased MTM level was associated with shorter overall survival during 3-year follow-up (*P*<0.05, Figure 1C). Univariate cox regression analysis identified that tumor size (*P*<0.01), invasive depth (*P*<0.05), lymphatic metastasis (*P*<0.05), TNM stage (*P*<0.01) and MTM expression (*P*<0.05) were prognostic factors. Though MTM expression was not a statistically significant independent predictor of patient survival by multivariate analysis, higher MTM expression indicated better survival for GC patients (HR=0.153, *P*=0.069) (Table 2).

**Table 2:** Univariate and multivariate cox regression analyses for overall survival in 76 patients with gastric cancer

Variable	Univariate Cox regression		Multivariate Cox regression	
	HR	*P* value	HR	*P* value
**Age**				
<60	1		1	
≥60	1.444	0.451	1.294	0.610
**Gender**				
Male	1		1	
Female	0.97	0.947	1.026	0.959
**Tumor size**				
<5cm	1		1	
≥5cm	7.087	0.002^**^	8.827	0.001^**^
**Differentiation**				
Poor	1		1	
Moderate/High	0.732	0.522	0.633	0.368
**Invasive depth**				
T1-2	1			
T3-4	51.64	0.034^*^		
**Lymphatic metastasis**				
N0	1			
N1-N3	59.454	0.026^*^		
**Distant metastasis**				
M0	1		1	
M1	1.955	0.369	4.079	0.098
**Stage (TNM)**				
I-II	1			
III-IV	20.025	0.004^**^		
**MTM expression**				
Low	1		1	
High	0.122	0.040^*^	0.153	0.069

^*^P<0.05; ^**^P<0.01.

### Overexpression of MTM suppresses cell migration and invasion *in vitro*

**Figure 2:**
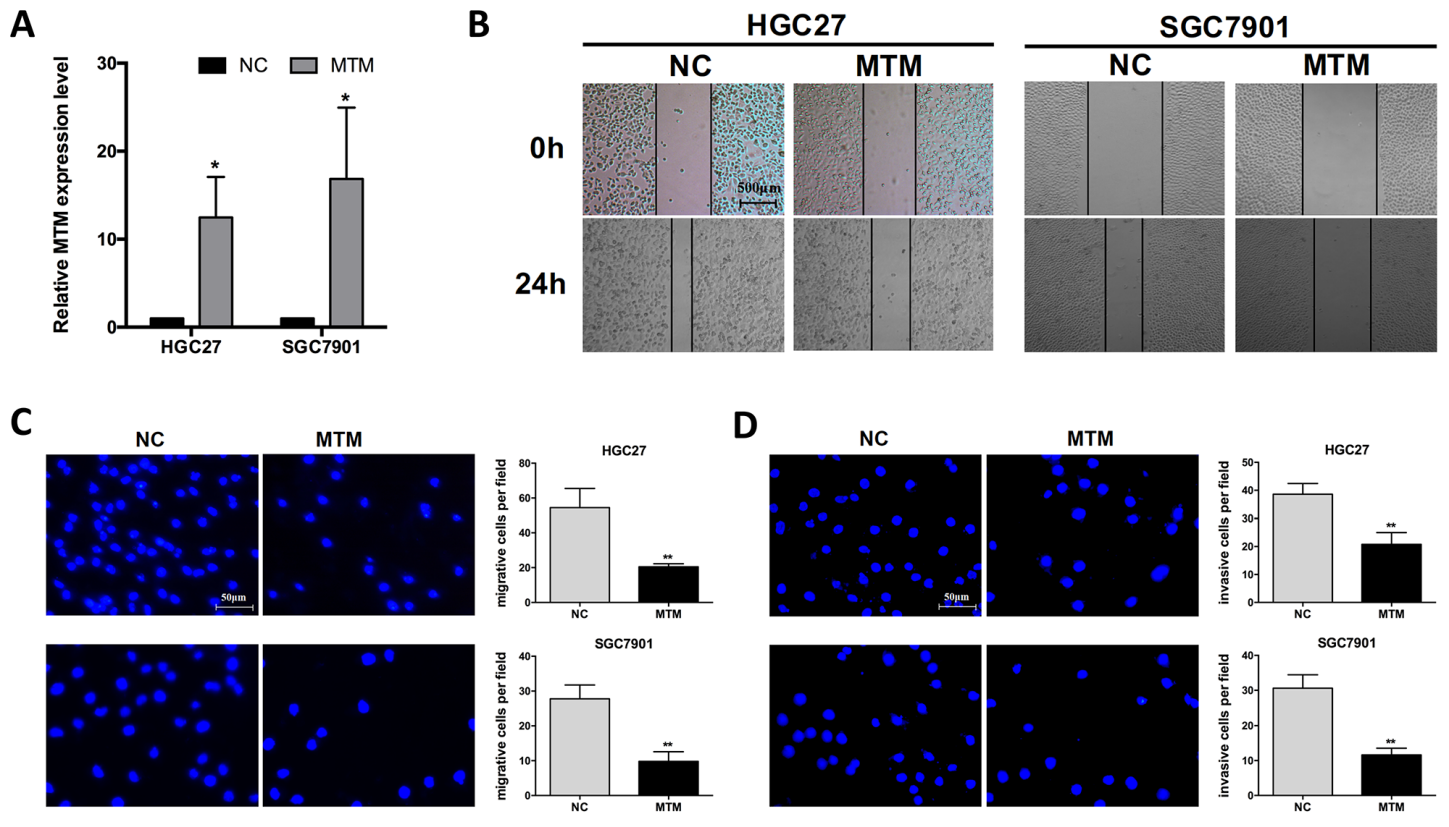
Overexpression of MTM inhibited the migration of gastric cancer (GC) cells **(A)** Transfection with pcDNA3.1-MTM markedly increased MTM expression in GC cells. Data were presented as the mean ± SD (n=3, ^*^P<0.05). **(B, C)** Impact of MTM overexpression on the migration of GC cells by wound healing assay (B) and by transwell migration assay (C). Data were presented as the mean ± SD (n=3, ^**^P<0.01). **(D)** Impact of MTM overexpression on the invasion of GC cells by transwell invasion assay. Data were presented as the mean ± SD (n=3, ^**^P<0.01).

To explore the functional involvement of MTM in GC carcinogenesis, two GC cell lines (HGC27 and SGC7901) were selected to overexpress MTM for the subsequent experiments (Figure 2A). Wound healing assays showed the weakened motility in HGC27 and SGC7901 cells with enhanced MTM expression (Figure 2B). Transwell assay also confirmed that overexpression of MTM significantly inhibited the cell migration of HGC27 and SGC7901 cells by 62.4% and 60.4%, respectively, when compared to controls (Figure 2C). Further invasion assay revealed that overexpression of MTM inhibited HGC27, SGC7901 cell invasion by 46.1% and 62.1%, respectively (Figure 2D).

### Effect of MTM overexpression on GC cell proliferation

**Figure 3:**
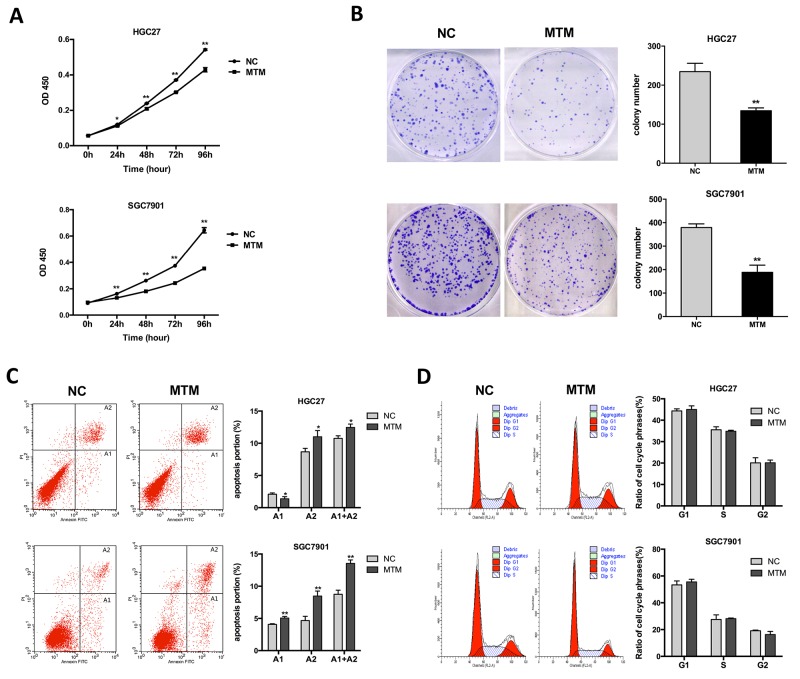
Effects of MTM overexpression on gastric cancer (GC) cell proliferation **(A)** CCK-8 assays showed the proliferation of GC cells was inhibited by MTM overexpression. Data are represented as mean ± SD (n=5, ^*^P<0.05, ^**^P<0.01). **(B)** Representative pictures of colony formation in MTM-overexpressing GC cells. The histogram showed the average number of the survival clones. Data were presented as the mean ± SD (n=3, ^**^P<0.01). **(C)** The apoptotic rates of cells were detected by flow cytometry in transfected GC cells (A1, early apoptosis; A2, late apoptosis). Data represented the mean ± SD (n=3, ^*^P<0.05, ^**^P<0.01). **(D)** Cell cycle distribution was analyzed by flow cytometry in GC cells. Data were presented as the mean ± SD (n=3, all *P*>0.05).

Next, we examined the effect of MTM overexpression on GC cell proliferation by using CCK-8 assays. GC cells overexpressing MTM showed a significant decrease in cell proliferation compared with negative controls (Figure 3A). The similar effects were also observed in colony formation assays where the colony numbers were decreased following the overexpression of MTM (Figure 3B). These findings suggest that ectopic expression of MTM suppresses GC cell proliferation.

To assess potential mechanisms of MTM in GC cell proliferation, cell cycle and apoptosis assays were performed by flow cytometry. Both HGC27 and SGC7901 cells overexpressing MTM had a significant increase in the proportion of late apoptotic cells versus negative controls (Figure 3C). However, no significant difference was found in the change of the cell cycle distributions (Figure 3D). Thus, these findings indicate that MTM overexpression does not affect cell cycle arrest but drives late apoptosis, which may lead to inhibition of cell proliferation.

### MTM and MT1F are coordinately expressed in GC

**Figure 4:**
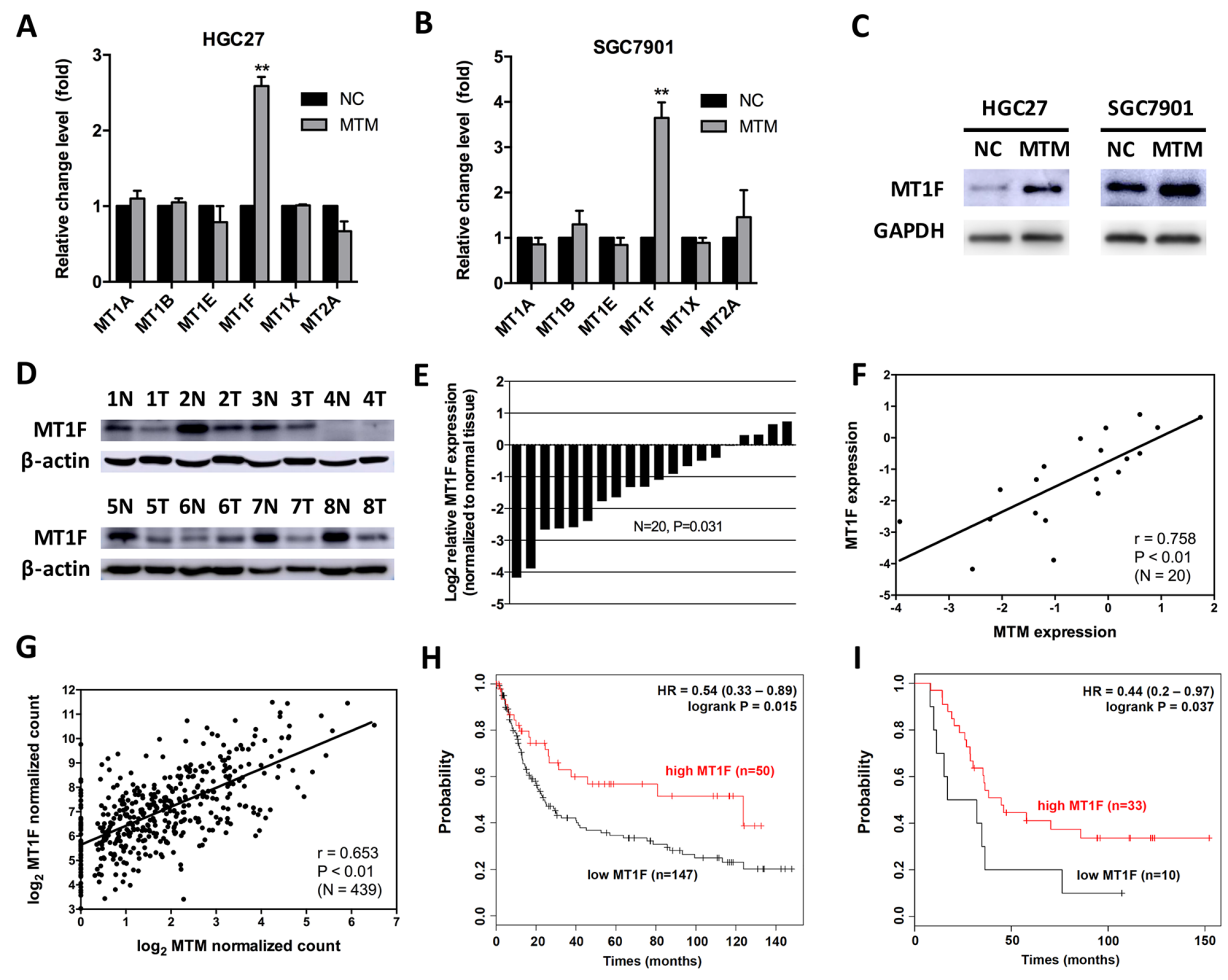
MTM and MT1F are coordinately expressed in gastric cancer (GC) **(A, B)** The mRNA levels of 6 MTs in MTM-overexpressing GC cells. Data are presented as the mean ± SD (n=3, ^**^P<0.01). **(C)** The protein level of MT1F in MTM-overexpressing GC cells. **(D)** The protein level of MT1F in 8 representative matched normal gastric mucosa -GC tissue pairs (N: normal, T: tumor). **(E)** MT1F was significantly decreased in 20 GC tissues. Expression levels are shown as log2-fold change to matching normal gastric mucosa tissues. **(F)** Expression levels of MTM and MT1F were significantly correlated in GCs (n=20, r=0.815, *P*<0.01). **(G)** Correlation of MTM and MT1F mRNA levels in human GCs from TCGA dataset (n=439, r=0.653, *P*<0.01). **(H)** Kaplan-Meier curves for relapse-free survival of GC patients classified by MT1F expression (GEO: GSE15459). **(I)** Kaplan-Meier curves for relapse-free survival of GC patients classified by MT1F expression (GEO: GSE22377).

MTs protein genes have been recognized as related to heavy metal detoxification and protection against oxidative stress and cancer [17]. To investigate whether there is a link between MTM and MTs protein genes in GC, we firstly measured 6 MTs expression in HGC27 and SGC7901 cells overexpressing MTM. MT1F mRNA level was remarkably upregulated in response to MTM overexpression, while no significant alteration was found in other MTs genes, including MT1A, MT1B, MT1E, MT1X and MT2A, and western blot assay confirmed the coordinated expression pattern between MTM and MT1F at protein levels (Figure 4A–4C).

Then, we measured the mRNA and protein levels of MT1F in GC tissues that examined for MTM. As expected, the protein level of MT1F was down regulated in the majority of GCs (Figure 4D). The mRNA level of MT1F showed the same trend (16/20, average fold change 0.427, *P*<0.01; Figure 4E), and was strongly correlated with MT1F in these tissues (Pearson correlation coefficient r=0.758, *P*<0.01; Figure 4F). We also used published human GC datasets (493 GC patients from TCGA) to validate the correlation between MTM and MT1F expression (Pearson correlation coefficient r=0.653, *P*<0.01; Figure 4G). Importantly, Kaplan-Meier plotter analysis of published dataset [18] (www.kmplot.com) found that high expression level of MT1F could identify patients with an improved probability of relapse-free survival, supporting a tumor suppressive role of MT1F in GC (GSE15459: HR=0.54, [95% CI 0.33–0.89], Figure 4H; GSE22377: HR=0.44, [95% CI 0.2–0.97], Figure 4I).

### Overexpression of MT1F inhibits GC cell migration and invasion

**Figure 5:**
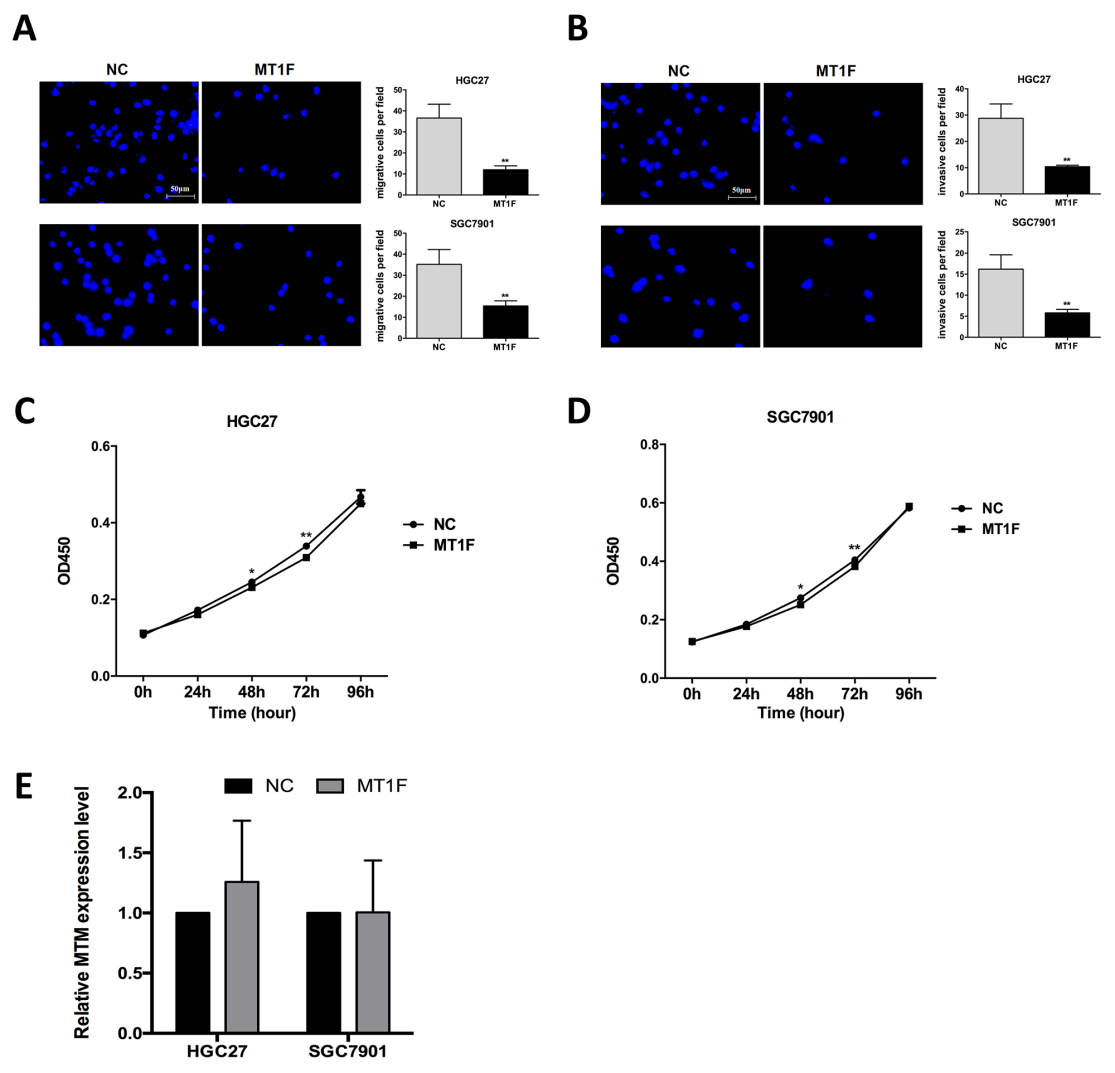
Overexpression of MT1F inhibits gastric cancer (GC) cell motility, but does not influence cell proliferation **(A)** Impact of MT1F overexpression on the migration of GC cells. Data were presented as the mean ± SD (n=3, ^**^P<0.01). **(B)** Impact of MT1F overexpression on the invasion of GC cells. Data were presented as the mean ± SD (n=3, ^**^P<0.01). **(C, D)** The proliferation of GC cells was not influenced by MT1F overexpression. Data are represented as mean ± SD (n=5, ^*^P<0.05, ^**^P<0.01). **(E)** MT1F Overexpression in GC cells did not alter MTM expression. Data are represented as mean ± SD (n=3, all *P*>0.05).

To our knowledge, the biological function of MT1F in GC has not yet been reported. Here, we investigated that decreased expression of MT1F mRNA and protein was found in 7 GC cell lines compared with GES-1 (Supplementary Figure 1). Overexpression of MT1F impaired the ability of cell migration and invasion in HGC27 and SGC7901 cells (Figure 5A, 5B). However, there were no significant differences in cell proliferation between MT1F-overexpressing cells and controls (Figure 5C, 5D). In order to elucidate the relation between MT1F and MTM, we detected the MTM level in MT1F-overexpressing cells. However, MT1F overexpression failed to significantly alter MTM *in vitro* (Figure 5E).

### Knockdown of MT1F in the MTM-overexpressing GC cells restored cell migration and invasion

**Figure 6:**
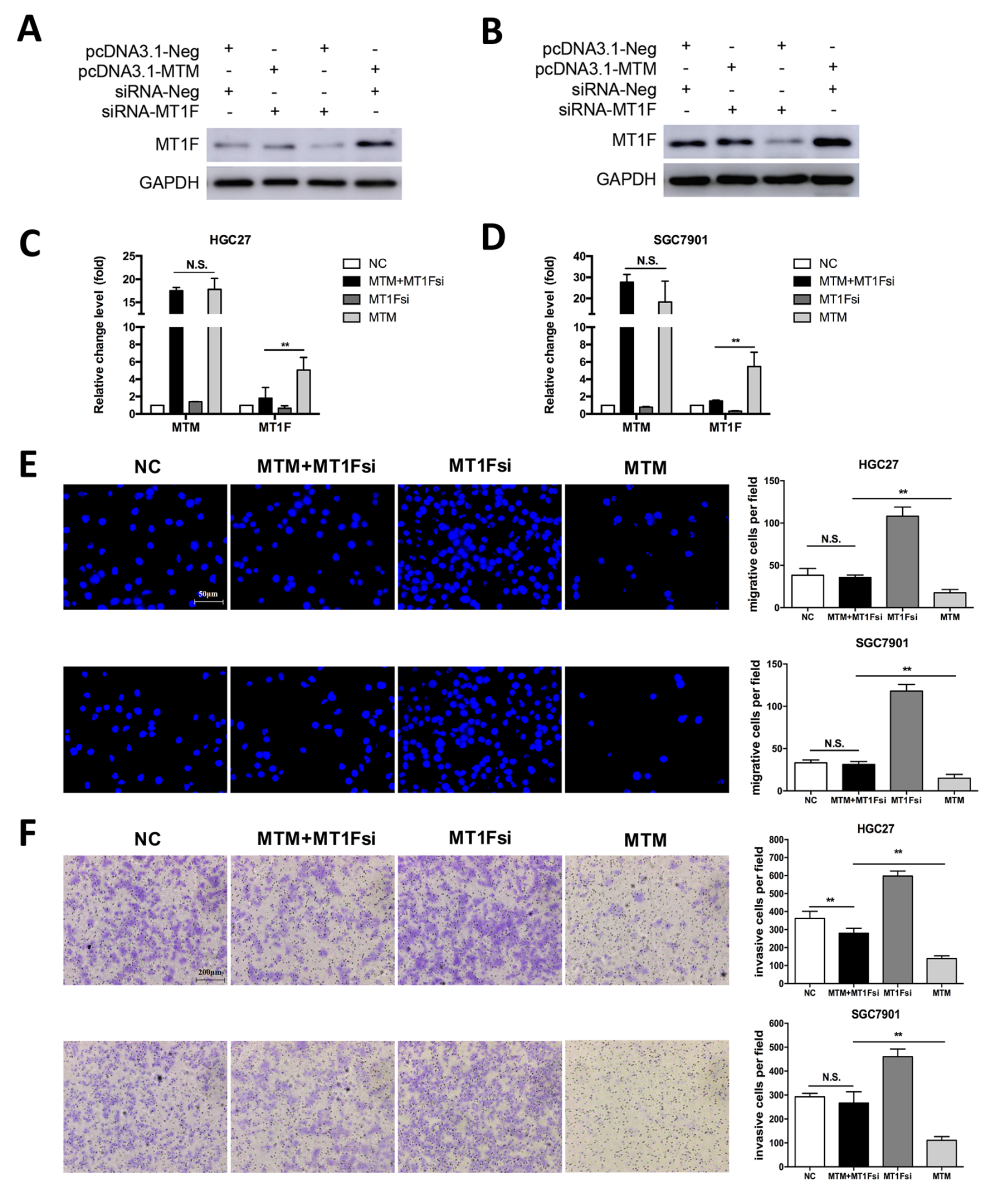
Knockdown of MT1F restores cell migration and invasion abilities in MTM-overexpressing cells *in vitro* **(A, B)** MT1F protein expression in HGC27 cells (A) and SGC7901 cells (B) after transfection with control vector or pcDNA3.1-MTM or control siRNA or siRNA against MT1F. **(C, D)** The expression of MTM in GC cells after transfection with control vector or pcDNA3.1-MTM or control siRNA or siRNA against MT1F. Data were shown as the mean ± SD (n=3, ^**^P<0.01). **(E)** Knockdown of MT1F Knockdown restored cell migration in MTM-overexpressing GC cells. Data were presented as the mean ± SD (n=3, ^**^P<0.01). **(F)** MT1F Knockdown restored cell invasion in MTM-overexpressing GC cells. Data were presented as the mean ± SD (n=3, ^**^P<0.01).

**Figure 7:**
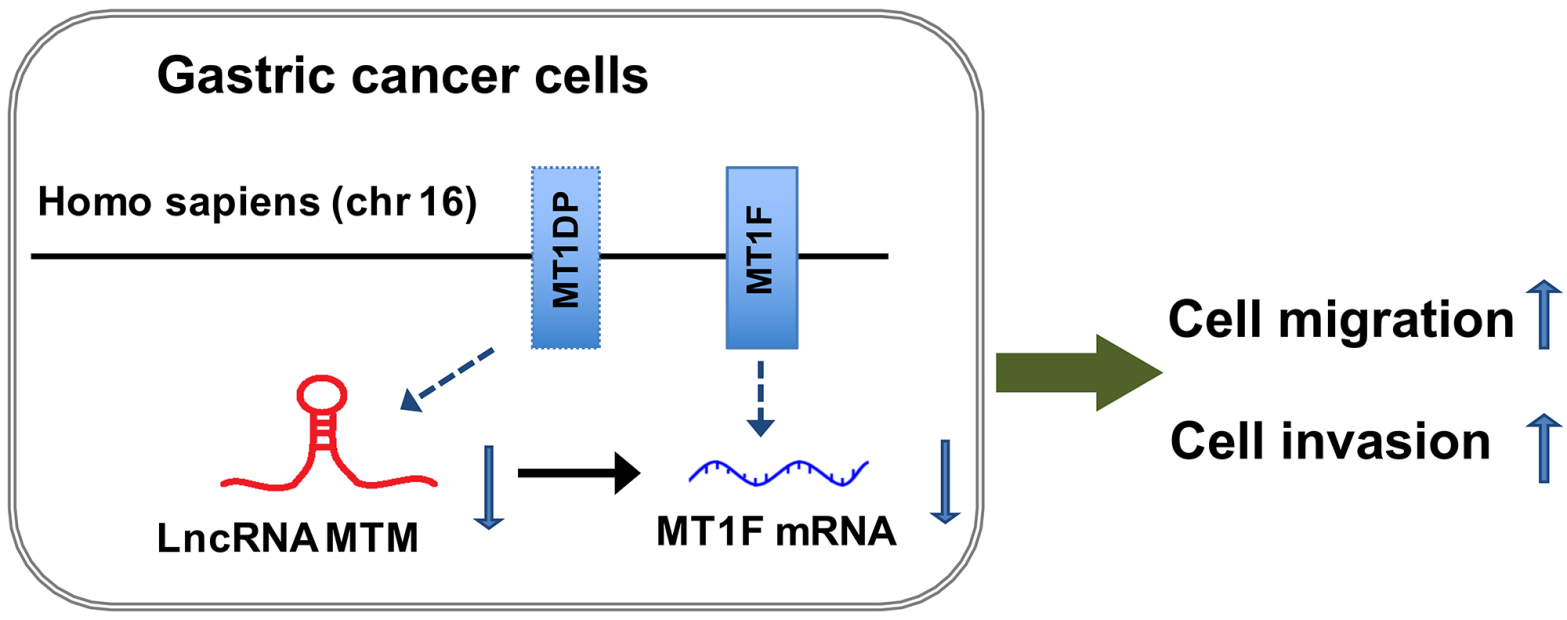
Possible mechanisms underlying the functional involvement of MTM and MT1F in gastric cancer (GC) cells MTM, a lncRNA transcribed from MT1DP gene, is located at chromosome 16 (Homo sapiens). Decreased expression of MTM has impact on its nearby protein-coding gene MT1F and inhibits the expression of MT1F at transcriptional level, resulting in enhanced ability of GC cell migration and invasion.

As MTM induced MT1F transcription was observed in MTM-overexpressing GC cells, we determined whether reduced motility in MTM-overexpressing cells is due to increased MT1F levels. MT1F specific siRNA was used to knockdown MT1F in HGC27 and SGC7901 cells overexpressing MTM (Figure 6A–6D). Decreasing the levels of MT1F in cultured MTM-overexpressing cells restored their migration and invasion, at least in part, to the level found in control cells (Figures 6E, 6F). Thus, MT1F is a key target of MTM in GC cells. Collectively, our results indicate that decreased expression of MTM may inhibit the expression of MT1F at transcriptional level and results in enhanced ability of GC cell migration and invasion (Figure 7).

## DISCUSSION

Increasing evidence established the participation of lncRNAs in gastric carcinogenesis. Examples include promotion of cell invasion and metastasis by HOTAIR [19], control of cell apoptosis by GAS5 [20], modulation of metastasis via epithelial–mesenchymal transition by SPRY4-IT1 [21], and regulation of cell growth and apoptosis by H19 in GC [22]. Though many new functions have been ascribed to these RNA species, the functional roles of most of these transcripts in GC remain unclear.

In this study, we focused on lncRNA MTM and explored its correlation with GC. We observed that MTM was down regulated in GC cell lines and tissues. Specifically, decreased expression of MTM in GCs was associated with more advanced clinical staging, deeper invasion and increased lymphatic metastasis. In addition, we found that decreased MTM level was associated with shorter overall survival and MTM expression might be an important prognostic factor for GC patients. Further investigations showed that ectopic expression of MTM resulted in diminished cell migration, invasion, proliferation and increased apoptosis in GC cells. These findings demonstrate that MTM may act as a candidate tumor suppressor in GC. To our knowledge, the decreased expression of lncRNA MTM may due to transcriptional repression by some transcription factors, inhibitive regulation by microRNA or through promoter hypermethylation in GC tissues. However, the exact mechanisms are still being investigated.

Unlike the mechanism of microRNA (miRNA) silencing target genes via complementary base pairing with mRNAs, lncRNAs can control local or global gene expression by diverse and complex means. However, the molecular mechanisms of lncRNAs cannot be inferred directly from sequence or structure with the diversity of lncRNAs. One emergent theme is the involvement of lncRNAs in regulating the expression of nearby protein-coding genes [23, 24]. Given the close proximity of MTM to MTs, we hypothesized that MTM could exert its biologic effects via MTs modulation. We demonstrated that MT1F exhibited similar expression pattern with MTM in both GC cell lines and tissues. Interestingly, MT1F is a MT family protein-coding gene whose transcriptional start site occurs approximately 14kb 5’ to that of MTM. The fact that MT1F mRNA and protein level was enhanced after overexpression of MTM suggested that MT1F might represent an important downstream effector of MTM.

Yan DW et al [25] demonstrated that MT1F gene expression was significantly decreased in colon cancer tissues through mechanism by loss of heterozygosity (LOH) and exogenous MT1F expression increased RKO cell apoptosis, inhibited RKO cell migration and invasion. The mechanisms underlying MT1F induced alteration in cancer is largely unknown. We found that MT1F overexpression resulted in impaired GC cell motility. MT1F knockdown in the MTM-overexpressing GC cells restored cell migration and invasion. Taken together, it is likely that decreased MTM inhibited GC cell migration and invasion by suppressing the expression of MT1F at transcriptional level. However, further investigations are required to explore the underlying molecular mechanisms.

In summary, we identified that MTM was downregulated in GC and overexpression of MTM inhibited GC cell migration and invasion. We also provide evidence that MT1F may represent a downstream effector of MTM, thus establishing an example of transcriptional regulation between lncRNA and protein-coding genes in GC.

## MATERIALS AND METHODS

### Patients and sample collection

A total of 92 patients (64 men, 28 women) with GC were enrolled in this study. The diagnosis of GC was histopathologically confirmed. No patient received pre-operative treatment. Tumor tissues and adjacent paired normal gastric mucosa (NGM) tissues, 5 cm from the edge of tumors, were immediately frozen in liquid nitrogen when resected, and stored at −80°C until use. Clinicopathological characteristics collected on all subjects include age, gender, and GC features such as tumor size, differentiation, histologic stage, invasive depth, the status of lymphatic metastasis and distant metastasis. Staging of GC was evaluated on the basis of the tumor-node-metastasis (TNM) classification system.

The study protocol was approved by the Clinical Research Ethics Committee of the Institute of Gastroenterology of Zhejiang University. Informed consent was obtained from all patients for the use of their tissues in this study.

### Cell line and culture conditions

The human GC cell lines (AGS, BGC823, HGC27, MGC803, SGC7901, MKN28 and MKN45) and 1 non-malignant gastric epithelial cell line (GES-1) were obtained from Riken Gene Bank (Tsukuba, Japan) and American Type Culture Collection (ATCC, Manassas, VA, USA). All of the cell lines were maintained in the recommended culture conditions and incubated at 37°C in a humidified environment containing 5% CO2.

### RNA extraction and qRT-PCR analyses

Total RNA was extracted from tissues or cultured cells with TRIzol reagent (Invitrogen Life Technologies) according to the manufacturer’s protocols. RNA reverse transcribed to cDNA from 1μg of total RNA in a final volume of 20 μl using a Reverse Transcription Kit with gDNA Eraser (Takara). The expression level of MTM was determined with quantitative RT-PCR (qRT-PCR) by using SYBR Premix Ex Taq II Kit (TaKaRa) in a LightCycler480 System. PCR were repeated in triplicate. Glyceralde- hyde-3-phosohate dehydrogenase (GAPDH) was used as an endogenous control in cell samples and snRNA U6 in tissue samples. The relative expression level of MTM and MT1F in GC tissues was measured by the 2^-ΔΔ*C*t^ methods. The primer sequences were shown in Supplementary Table 1.

### Western blot assays

Cells were lysed with RIPA protein extraction reagent (Beyotime, Beijing, China) supplemented with a protease inhibitor cocktail (Roche, CA, USA). Cells were harvested 48 hours after expression plasmid transfection. We used 1:1000 anti-human metallothionein Mouse monoclonal antibody (ab12228; abcam, UK) and 1:10000 anti-human GAPDH rabbit monoclonal antibody (ab181602; abcam, UK). GAPDH was used as an endogenous control.

### Transfection of GC cells

Expression plasmid for MTM was constructed by cloning of the full-length MTM into the mammalian expression vector pcDNA3.1 (+) with BamHI and EcoRI restriction enzyme sites, while MT1F was cloned into pcDNA3.1 (-) with XhoI and KpnI restriction enzyme sites. The full-length products were amplified by RT-PCR using specific primers (Supplementary Table 1). The sequences were confirmed by DNA sequencing. siRNAs for the human MT1F and the negative control olignucleotides were purchased from GenePharma (GenePharma, Shanghai, China). Cells were cultured in six-well plates for 24 h and then transfected with pcDNA3.1-MTM, pcDNA3.1-MT1F or si-MT1F by using Lipofectamine 3000 (Invitrogen, Carlsbad, CA, USA) according to the manufacturer’s instructions. Cells were harvested after 72h for qRT-PCR and western blot analyses.

### Wound-healing assay

Cells transfected with pcDNA3.1 vector or pcDNA3.1-MTM were seeded onto the six-well plates and then scratched with a p10 pipette tip to create a gap. The wells were rinsed with PBS to remove displaced cells and fresh media without serum was added. The randomized images of the scratched areas were taken (#x00D7;40 magnification) over 0h and 24h.

### Cell migration and invasion assays

Cell migration was assessed by modified Boyden transwell chambers assay (Corning, NY, USA). Briefly, 5 #x00D7; 10^4^ cells/well were plated into 200 μL of 1% FBS medium in the upper chamber, and 600 μL of medium containing 10% FBS were added to the lower chamber. The cells were incubated for 18 h. The cells on the bottom of the membrane were fixed and stained with DAPI and the cells that did not migrate through the pores of the membrane were manually removed with a cotton swab. For cell invasion assay, Matrigel-coated transwell chambers were prepared 6 hours before the seeding of cells. 1.5 #x00D7; 10^5^ cells/well were plated into 200 μL of 1% FBS medium in the upper chamber with 600 μL of 10% FBS medium in the lower chamber. After 24 hours, the cells and Matrigel in the upper inserts were discarded and the cells on the bottom of the membrane were fixed and stained with DAPI or crystal violet (Sigma-Aldrich, St. Louis, MO, USA). The number of cells was then counted by fluorescence microscope.

### Cell proliferation assays

Cells transfected with pcDNA3.1 vector or pcDNA3.1-MTM was used for cell proliferation assays. 48 hours after transfection, 3000 cells per well were seeded onto the 96-well plates. After 6 h of culture (day 0), as well as at 24h (day 1), 48h (day 2), 72h (day 3) and 96h (day 4), cell proliferation was measured using Cell counting kit-8 (CCK-8) assays (Dojindo, Japan) according to the manufacturer’s protocols.

### Colony formation assay

Cells were trypsinised into single-cell suspension 48 h after transfection. 1000 cells were plated into each well of a 6-well plate. Colonies were fixed with methanol and stained with 0.1% crystal violet in PBS for 30 min. The colony formation was determined by counting the number of stained colonies.

### Cell apoptosis and cell cycle analysis

Cell apoptosis assays were performed using the FITC Annexin V Apoptosis Detection Kit I (BD Biosciences) by flow cytometry analysis (FCA). Cell cycle analysis was detected by the Cell cycle staining solution kit (MultiSciences, China). The percentages of the cells in G0–G1, S, and G2/M phases were counted and compared.

### Statistical analysis

All statistical analyses were performed using SPSS 20.0 software (IBM, SPSS, Chicago, IL, USA). For cell biological assays, each experiment was repeated at least thrice, unless otherwise stated. The significance between two comparable groups was calculated by two-tailed Student’s *t* test. Different expression levels of MTM or MT1F between the tumor tissues and the paired adjacent normal tissues were estimated by Mann–Whitney U test. Correlation between MTM and MT1F mRNA expression was analyzed using Pearson correlation test. The survival curve was estimated by Kaplan-Meier method and log-rank test. The univariate and multivariate cox regression analysis were performed to evaluate the prognostic factors of GC patients. *P*<0.05 was considered statistically significant.

## SUPPLEMENTARY MATERIALS FIGURE AND TABLE


