# Supplementary material for: Decreased long non-coding RNA MTM contributes to gastric cancer cell migration and invasion via modulating MT1F
Source: Oncotarget. 2017 Oct 26;8(57):97371–83. doi: 10.18632/oncotarget.22126 (PMC5722569; doi:10.18632/oncotarget.22126)
Supplement: Supplementary file 1 [file oncotarget-08-97371-s001.pdf]

Decreased long non-coding RNA MTM contributes to gastric cancer cell migration and invasion via modulating MT1F

SUPPLEMENTARY MATERIALS

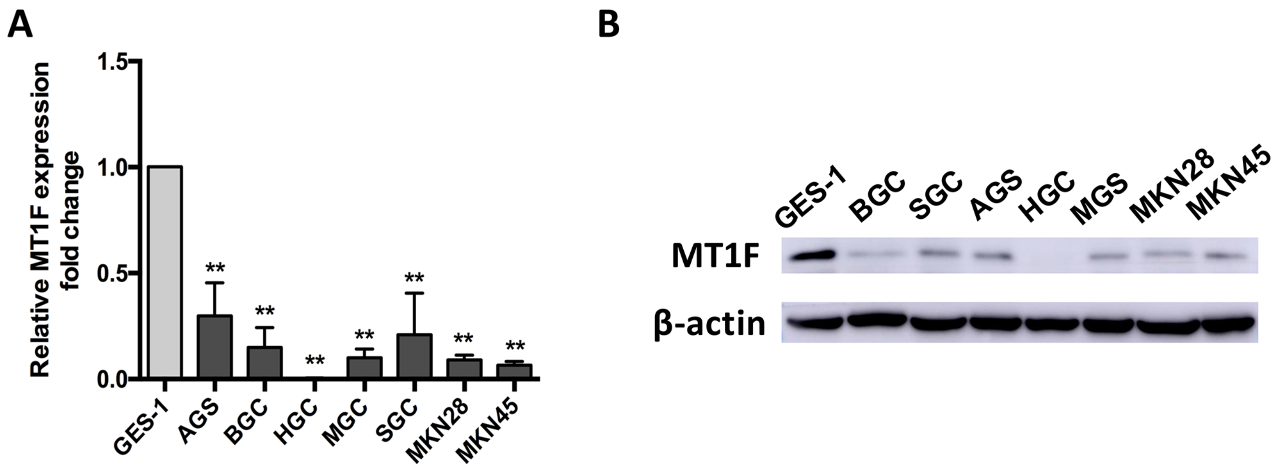

Supplementary Figure 1: MT1F expression is down regulated in human gastric cancer (GC) cell lines. **(A)** Expression of MT1F mRNA in 7 GC cell lines and GES-1. Expression levels are normalized to GES-1. Data were presented as the mean ± SD (n=3, **P<0.01). **(B)** The protein expression level of MT1F in 7 GC cell lines and GES-1.

Supplementary Table 1: The sequences of the primers used in the study

Primers	Sequence, 5'–3'
**Primers for qRT-PCR:**	
MTM-1F	CTCCAGCCTCACCTGATCTC
MTM-1R	AGCTGCACTTCTCCAATGC
MT1A-F	GGGCATCAGAGAAGTGCAG
MT1A-R	ATGGGTCAGGGTTGTATGGA
MT1B-F	GAACTCCAGGCTTGTCTTGG
MT1B-R	GGTACATTTGCACTCTTTGCAC
MT1E-F	CTGCTTGTTCGTCTCACTGG
MT1E-R	GCATTTGCACTCTTTGCACT
MT1F-F	GACTGATGCCAGGACAACCT
MT1F-R	AGGAATGTAGCAAATGGGTCA
MT1X-F	GGGAACTCCTGCTTCTCCTT
MT1X-R	GCATTTGCACTCTTTGCATT
MT2A-F	GACTCTAGCCGCCTCTTCAG
MT2A-R	GCATTTGCACTCTTTGCATT
GAPDH-F	GAAGGTGAAGGTCGGAGT
GAPDH-R	GAAGATGGTGATGGGATTTC
snRNA U6-F	CTCGCTTCGGCAGCACA
snRNA U6-R	ATTTA TTTAT GGGGA TTTGG GTTT
MMP2-F	ACGACCGCGACAAGAAGTAT
MMP2-R	ATTTGTTGCCCAGGAAAGTG
MMP7-F	GTCTCTGGACGGCAGCTATG
MMP7-R	GAGCCTGTTCCCACTGTAGC
MMP9-F	TCGAACTTTGACAGCGACAAG
MMP9-R	GCACTGAGGAATGATCTAAGC
MMP14-F	GGCGGGTGAGGAATAAC
MMP14-R	AGCATCAATCTTGTCGGTAG
**Primers for full-length: product:**	
MTM-2F	CGGGATCCCCTGTGGCTTAGGAACTC
MTM-2R	CGGAATTCAAGGCCAACAATGTTTATTATC
MT1F-F	ACGGGCCCTCTAGACTCGAGCGCCAC CATGGACCCCAACTGCTCCTGCGCCGC
MT1F-R	TTAAACTTAAGCTTGGTACCTCAGTCGC AGCAGCTGCACTTC

-F: forward primer, -R: reverse primer.
